# Comparison of donor hearts from hypoxic cardiac arrest followed by warm ischemia and from exsanguination as non-beating donor models in rat heart transplantation

**DOI:** 10.1186/1749-8090-10-S1-A196

**Published:** 2015-12-16

**Authors:** Shiliang Li, Sevil Korkmaz-Icöz, Tamás Radovits, Péter Hegedűs, Matthias Karck, Gábor Szabó

**Affiliations:** 1 Department of Cardiac Surgery, University of Heidelberg, 69120 Heidelberg, Germany; 2 Heart and Vascular Center, Semmelweis University, 1122 Budapest, Hungary

## Background/Introduction

Non-heart-beating donation, in the situation where a patient cannot be diagnosed as dead using brainstem criteria, may be an alternative to overcome shortage of donors. Preclinical studies showed that functional recovery of hearts after circulatory determination of death (DCDD) were similar to that of hearts from brain-dead donor. However, there are several experimental methods of simulating DCDD conditions such as exsanguination and hypoxic cardiac arrest both followed by warm ischemia.

## Aims/Objectives

We aimed to compare two non-heart beating models in rat heart transplantation in which grafts were retrieved under exsanguination- or apnea-induced non-heart-beating conditions.

## Method

Donor hearts were either left in situ for 10 min after cardiac arrest, which was induced by rapid exsanguination (exsanguination group, n = 6) or subjected to hypoxic cardiac arrest followed by 10 min of warm ischemia (agonal apnea group, n = 6). Additionally, a control group, in which heart grafts were retrieved from heart beating donors, was used (n = 6). Then, hearts were perfused with a cold preservation solution (Custodiol), explanted, stored at 4°C in Custodiol for 1h and heterotopically transplanted. We evaluated left-ventricular graft function and assessed protein expression 1.5h after transplantation.

## Results

After transplantation, significantly decreased systolic function (left-ventricular systolic pressure: exsanguination 64 ± 6 mmHg vs. apnea 47 ± 6 mmHg vs. control 89 ± 5 mmHg; developed pressure: exsanguination 62 ± 6 mmHg vs. apnea 43 ± 6 mmHg vs. control 88 ± 6 mmHg; dP/dtmax: exsanguination 1700 ± 85 mmHg/s vs. apnea 2313 ± 262 mmHg/s vs. control 3333 ± 147 mmHg/s, p < 0.05) was observed in both exsanguinations- and apnea-groups compared to controls. Additionally, apnea-group showed further impaired diastolic function when compared with the exsanguination-group (dP/dtmin: apnea 1044 ± 81 mmHg/s vs. exsanguination 1588 ± 160 mmHg/s, p < 0.05). Protein levels of caspase-3, tumor necrosis factor alpha, cyclooxygenase-2, nuclear factor KappaB, inducible nitric oxide synthase, cytochrome-c and SERCA-2 (assessed by Western Blot) did not differ between the groups.

## Discussion/Conclusion

Our results showed that both models appear to be useful for investigating functional and molecular characterization of potential use of hearts after DCDD.

